# Environmental Friendly Fabrication of Porous Cement Membranes via Reusable Camphene-Based Freeze-Casting Method

**DOI:** 10.3390/membranes12090867

**Published:** 2022-09-08

**Authors:** Zhen Wang, Xiaojuan Wang, Zhantong Sun, Xiaofeng Wang, Hongdong Wang, Congjie Gao, Xueli Gao

**Affiliations:** 1 Frontiers Science Center for Deep Ocean Multispheres and Earth System, Key Laboratory of Marine Chemistry Theory and Technology, Ministry of Education, College of Chemistry and Chemical Engineering, Ocean University of China, Qingdao 266100, China; 2 SEPCOIII Electric Power Construction Co., Ltd., Qingdao 266100, China

**Keywords:** porous cement membrane, separation performance, camphene, freeze casting, reusable

## Abstract

Inorganic membranes have been developed rapidly in recent years because of excellent anti-fouling performance, high mechanical strength and outstanding resistances to acid and alkali. However, the high production cost still restricts its large-scale industrial application. In this work, an environmental friendly unidirectional freezing method via introducing camphene as a reusable template was adapted to prepare porous cement membranes (PCMs). The naturally formed and highly aligned porous structures of PCMs could be divided into three parts: a dense layer, a transition layer and a supporting layer. With the solid content rising from 40 wt.% to 60 wt.%, the pore size of the PCMs decreased from 3.34 nm to 3.62 nm, the bovine serum albumin (BSA) rejection increased from 81.3% to 93.5% and water flux decreased from 346.8 L·m^−2^·h^−1^ to 167.3 L·m^−2^·h^−1^ (0.2 MPa). Significantly, the performance of PCMs was maintained; even the camphene was reused 20 times. Additionally, the recovery rate of camphene could be reached up to 97.16%. Therefore, this method is cost effective and environmental friendly, which endowed the PCMs great potential in water treatment.

## 1. Introduction

With the rapid development of inorganic membranes, they have been used in many industries because of their good tolerance to organic solvents, high temperature and chemical corrosion, as well as high mechanical strength [1,2,3,4,5]. Especially in the field of water treatment, inorganic membranes have been used widely in recent years. For example, Lee and Cho [6] removed natural organic matter from drinking water with ceramic membranes. The ceramic membranes showed excellent performance in removing DBP precursors and they exhibited higher permeability than equivalent polymeric membranes. However, high energy consumption, complex preparation process and high material cost [7] have restricted the development and application of inorganic membranes. Cement, as a kind of conventional high-performance construction material, can be hardened into porous matrix easily through hydration reaction when exposed to water or humid environment [8] and, thus, has great potential as an inorganic membrane material. Compared with sintering, the hydration process is simpler, safer and more environmental friendly. Additionally, the cement is much cheaper than traditional inorganic membrane materials.

Compared with other methods, such as sol–gel [9] and phase inversion [10], the freeze-casting method can fabricate aligned porous [11], which is propitious to the water transport and the pore size can be controlled precisely. Generally, the freeze-casting technique consists of freezing the liquid suspension, followed by removing the pore precursors and, finally, obtaining solidified porous materials through sintering or other methods [12]. The method can produce unidirectional channels in the porous materials in the case of unidirectional freezing, where pores are the replica of the solvent crystals. For example, Abdullayev et al. [13] fabricated cement membranes with water as pore precursors and studied the effects of cooling rate and solid content on membrane properties. The water flux of cement membranes was about 181 L·m^−2^·h^−1^ under 0.5 bar when the cooling rate and solid content was 2 K/min and 50 wt.%. Dong et al. [7,14,15] explored the preparation of silicate cement compacts with functionally graded microstructure, including directional prismatic pore structure, lamellar structure and coralline structure, using the freeze-casting method. Cement-based porous materials with ultrafiltration membrane properties have been prepared via using tert-butyl alcohol (TBA) [7], water [14] and a mixture of TBA and water [15] as the pore precursors and the water flux could reach 460 L·m^−2^·h^−1^ [7] and 406 L·m^−2^·h^−1^ [15], respectively. The cement membranes exhibit significant advantages, such as adjustable pore structure, cheap raw materials and energy efficiency, but some problems, such as insufficient strength, still exist.

In this paper, the porous cement membranes (PCMs), which were used for water treatment, were prepared with camphene-based freeze casting under a mild condition. The highly aligned porous structures could be fabricated with the camphene as pore precursors, which was conducive to the penetration of water. The resulted membranes possessed strong compressive/flexural strength and superior separation performance because of the coralline structure. Moreover, camphene could be recycled easily because of its high melting point and then reused to prepare PCMs in this paper, while maintaining similar structure and performance compared to PCMs prepared with unused camphene, which is more consistent with the requirements of environmental protection. Further, the PCMs have great potential in the water treatment industry.

## 2. Materials and Methods

Commercially available silicate cement powder (P.O 42.5, d50 = 3.63 μm, Shanshui Group) was used as the basic material, while camphene (75%, containing 20% tricyclene, Aladdin) was used as the pore precursors. In addition, ethyl cellulose (99.5%, Sinopharm) and polyethylene glycol 400 (PEG-400, 99%, Sinopharm) were used as binder and dispersant, respectively.

Firstly, the cement (40–60 wt.%), camphene (37.5–57.5 wt.%), ethyl cellulose (0.5 wt.%), and PEG-400 (2 wt.%) were mixed using magnetic stirring for 6 h at 60 °C and then placed in a container to freeze for 1 h at −80 °C. Subsequently, the frozen raw material was freeze dried in a vacuum freeze-drying machine (FD-1A-80, Boyikang, Beijing, China) for 24 h [15] to remove the pore precursors and then cured in a humidor with 100% relative humidity at 25 °C for 28 d. Meanwhile, camphene could be recycled from the freeze-drying machine. To test permeation and separation of PCMs, the side of supporting layer was polished carefully and obtained cylindrical samples with thickness of about 5 mm and diameter of 5 cm. Finally, the PCMs were kept in water for subsequent testing.

**Figure 1:**
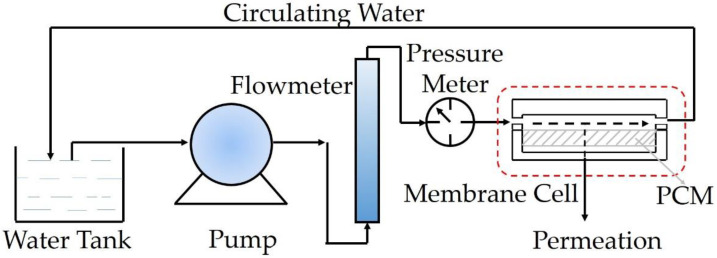
Diagram of crossflow filtration system for permeation and separation test.

The micromorphology and pore size of PCMs were characterized by scanning electron microscopy (SEM, HITACHI-4800, Tokyo, Japan) and automated surface and pore size analyzer (NOVA2200e, Quantachreme, Boynton Beach, FL, USA), respectively. The compressive and flexural strength of samples (12 mm diameter × 15 mm height) was measured using a bending and compression testing machine (DKZ-5000, TYE-300, Wuxi, Jianyi, China) along the freezing direction. The water flux, rejection of BSA (98%, Mw = 67 kDa, Sinopharm, Beijing, China) and polyethylene glycol 100,000 (PEG 100,000, 99%, Sinopharm) were measured with a crossflow filtration system (Figure 1) at 0.2 MPa. The concentration of BSA and PEG 100,000 in feed solution was 1000 mg/L and their concentration in permeation was tested by UV–vis spectroscopy (UV-2450, Shimadzu Co., Ltd., Kyoto, Japan) with an absorption wavelength at 280 nm and 510 nm, respectively, according to the Lambert–Beer law.

Pure water flux and BSA/PEG 100,000 rejection were calculated according to the following equation:

(1) $$F_{w}=\frac{V}{A\cdot t}$$

where *F_w_* is pure water flux, L·m^−2^·h^−1^; *V* is the volume of permeate water, L; *A* is the effective filtration area, m^2^; *t* is penetration time, h.

(2) $$R=\left(1-\frac{C_{p}}{C_{f}}\right)$$

where *R* is rejection, %; *C_p_* is the test molecule (BSA and PEG 100,000) concentration of permeation, g/L; *C_f_* is the test molecule concentration of feed solution, g/L.

All the membrane filtration performance tests were carried out 5 times and the average value was taken as final result to ensure accuracy in the results.

## 3. Results and Discussion

**Figure 2:**
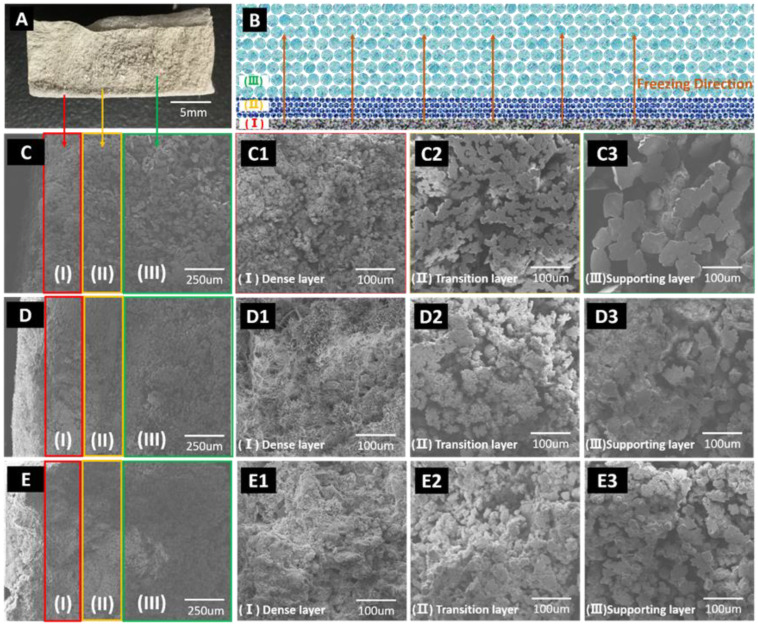
Typical PCM sample and micromorphology characterization. The overall image of PCM (**A**,**C**–**E**); the illustration of membrane cross-sectional microstructure (**B**); the SEM images of the gradient-pore-morphology of dense layer (**C1**–**E1**), transition layer (**C2**–**E2**) and supporting layer (**C3**–**E3**). The SEM images of PCMs with solid content at 40 wt.% (**C**,**C1**–**C3**), 50 wt.% (**D**,**D1**–**D3**) and 60 wt.% (**E**,**E1**–**E3**).

PCMs with gradient pore structure were prepared using the camphene-based freeze-casting method. As shown in Figure 2A–E, the PCMs could be divided into three layers, including a dense layer (Figure 2C1–E1), a transition layer (Figure 2C2–E2) and a supporting layer (Figure 2C3–E3), where the pore size of PCMs increased gradually along with the freezing direction. The dense layer endowed the PCMs with selectivity and the transition layer and supporting layer with micropores could reduce water transport resistance.

As can be seen from Table 1, along with the solid content increasing from 40 wt.% to 60 wt.%, the porosity and pore size of the dense layer decreased significantly from 65.4 vol.% to 47.4 vol.% and 3.62 nm to 3.34 nm, respectively. Meanwhile, the flexural strength and compressive strength increased from 8.6 MPa to 14.7 MPa and 15.6 MPa to 21.3 MPa separately. When solid content was relatively low, the rejection of solid particles to solidification fronts was so ineffective that camphene molecules diffused to the crystal surface easily, which led to bigger pore sizes. With the increase in solid content, the resistance of cement particles preventing camphene molecules from diffusing to the crystal surface enlarged, which led to smaller pore sizes [16]. Meanwhile, lots of heterogeneous nucleation sites were generated, resulting in uniform pore structure and high mechanical strength of PCMs [7]. In addition, with the solid content increasing from 40 wt.% to 60 wt.%, the water flux decreased from 346.8 L·m^−2^·h^−1^ to 167.3 L·m^−2^·h^−1^, while the BSA and PEG 100,000 rejection increased from 81.3% and 87.4% to 93.5% and 97.1%, respectively. This result is in accordance with the variation in membrane pore size, where a smaller pore size would lead to an increase in rejection and filtration resistance.

**Figure 3:**
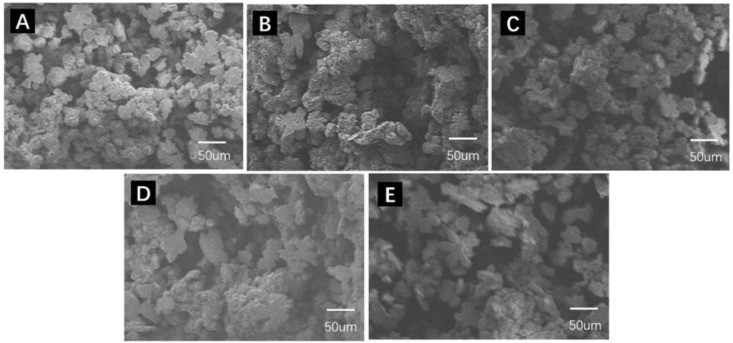
SEM images of PCMs’ supporting layer with different reused times of camphene under 60 wt.% solid content; (**A**–**E**) represent 0, 5, 10, 15 and 20 reuse times of camphene, respectively.

**Figure 4:**
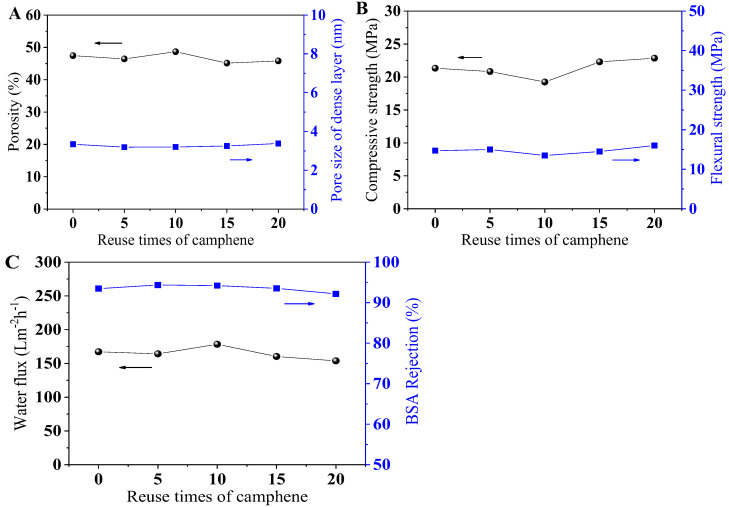
Properties of the PCMs prepared with different reuse times of camphene. Porosity and pore size of dense layer (**A**), compressive strength and flexural strength (**B**), water flux and BSA rejection (**C**) of PCMs with different reuse times of camphene.

Camphene was adhered to the cold trap wall during the freeze-drying process; thus, it could be recycled easily with a recovery rate up to 97.16% in the experiment. As seen in Figure 3, the microstructure of the PCMs prepared with reused camphene was almost identical, which suggested that the reused camphene had no influence on the pore structure of PCMs. The properties of PCMs prepared with recycled camphene are plotted in Figure 4. The porosity and average pore size of the dense layer are shown in Figure 4A and there was almost no change, indicating stability in the membrane pore structure. Figure 4B shows the relatively stable compressive and flexural strength of cement membranes, demonstrating the reused camphene had little effect on the mechanical strength of PCMs. The water flux and BSA rejection are shown in Figure 4C, exhibiting that the permeability and selectivity of PCMs were stable. In conclusion, the reused camphene had little effect on the properties of PCMs. Hence, camphene can be recycled in the preparation of PCMs, which is cost effective and environmental friendly.

## 4. Conclusions

This work showed that the camphene-based freeze-casting method could be used to prepare porous cement membranes with aligned pores. The method could prepare PCMs with three layers and control the pore size and physical properties effectively. Compared with the sintering process, the freeze-casting process and curing process are simple, energy saving and environmental friendly, which endues PCMs with great potential. Moreover, the performance of PCMs changes little with the increase in reuse times of camphene, which indicates that camphene can be recycled and the recovery rate of camphene could reach up to 97.16%.

## Figures and Tables

**Table 1:** Influence of solid content on the performance and pore structure of PCMs.

	Solid Content, wt.%		
	40	50	60
Porosity, %	65.4 ± 1.4	55.3 ± 2.1	47.4 ± 2.1
Pore size of dense layer, nm	3.62 ± 0.09	3.52 ± 0.06	3.34 ± 0.06
Compressive Strength, MPa	15.6 ± 0.4	18.5 ± 0.5	21.3 ± 1.6
Flexural Strength, MPa	8.6 ± 0.5	12.5 ± 0.4	14.7 ± 1.1
Water Flux, L·m^−2^·h^−1^	346.8 ± 5.1	228.0 ± 5.7	167.3 ± 5.5
BSA Rejection, %	81.3 ± 1.5	89.6 ± 1.8	93.5 ± 1.4
PEG 100,000 Rejection, %	87.4 ± 1.1	93.5 ± 0.9	97.1 ± 1.4

## Data Availability

Not applicable.
